# Primary Epstein–Barr virus infection shortly after primary *Cytomegalovirus* infection: a case report

**DOI:** 10.1186/s13256-021-02817-2

**Published:** 2021-05-03

**Authors:** Katsumasa Koyama, Takatoshi Anno, Takashi Urano, Ryo Shigemoto, Shintaro Irie, Fumiko Kawasaki, Miwa Kawanaka, Hirofumi Kawamoto, Hideaki Kaneto, Koichi Tomoda

**Affiliations:** 1 Department of General Internal Medicine 1, Kawasaki Medical School, 2-6-1 Nakasange, Kita-ku, Okayama, 700-8505 Japan; 2 Department of General Internal Medicine 2, Kawasaki Medical School, Okayama, 700-8505 Japan; 3 Department of Diabetes, Metabolism and Endocrinology, Kawasaki Medical School, Kurashiki, 701-0192 Japan

**Keywords:** Primary *Cytomegalovirus* infection, Primary Epstein–Barr virus infection, In a short period, Healthy adult Japanese woman

## Abstract

**Background:**

Infectious mononucleosis (IM) and mononucleosis-like illnesses are common viral infectious diseases which are often accompanied by a high fever, pharyngitis and lymphadenopathy in adults, although such infection in childhood is generally subclinical. Most cases of IM are caused by the Epstein–Barr virus (EBV) or *Cytomegalovirus* (CMV). However, it is difficult to diagnose IM only with subjective symptoms, and thus EBV and CMV are nearly indistinguishable in clinical practice.

**Case presentation:**

A 20-year-old healthy Japanese woman had a 2-day history of high fever and consulted us. She had sex for the first time 6 months earlier. Her virus antibodies showed that she was infected with primary CMV. About 5 months later, she again experienced high fever and lymph node enlargement at the posterior cervical region. Her virus antibodies showed that she was infected with primary EBV at that time.

**Conclusion:**

Herein, we report a healthy adult Japanese woman with primary EBV infection relatively soon after primary CMV infection. It is very interesting to compare the symptoms and/or clinical data after EBV and CMV infection in the same patient within a short period of time. Our patient was diagnosed based only on subjective symptoms, physical examination and laboratory data, without tests of such virus-related antibodies. Therefore, clinicians should bear in mind that primary EBV infection and/or primary CMV infection is possible when patients have symptoms such as high fever, pharyngitis and lymphadenopathy, even in healthy adults.

## Background

Infectious mononucleosis (IM) and mononucleosis-like illnesses (MLI) are common viral infectious diseases that are typically observed in childhood. After such infection, children are generally subclinical but adults often experience serious symptoms such as a high fever, pharyngitis and lymphadenopathy [1, 2]. Most cases of IM are caused by the Epstein–Barr virus (EBV) or *Cytomegalovirus* (CMV). It was reported that younger age, shorter interval from the onset to hospital visit, cervical lymphadenopathy, tonsillar white coat, hepatosplenomegaly or atypical lymphocytosis differentiates IM caused by EBV from that by CMV [3]. It was also shown that moderate elevation of lactate dehydrogenase and γ-glutamyl transpeptidase (γ-GTP) also differentiates IM caused by EBV from that by CMV [3]. In clinical practice, however, since it is difficult to diagnose IM and MLI only with subjective symptoms, EBV and CMV are nearly indistinguishable [4, 5]. In this context, serological testing for IM and MLI is important, but it requires several days to obtain the results. Moreover, EBV-specific antibody tests sometimes produce inaccurate results such as false negatives [6, 7] and cross-reactivity with CMV [8].

Herein, we report a healthy adult Japanese woman with primary EBV infection shortly after primary CMV infection. It is very interesting to compare the symptoms and/or clinical data after EBV and CMV infection in the same patient within a short period.

## Case presentation

**Fig. 1:**
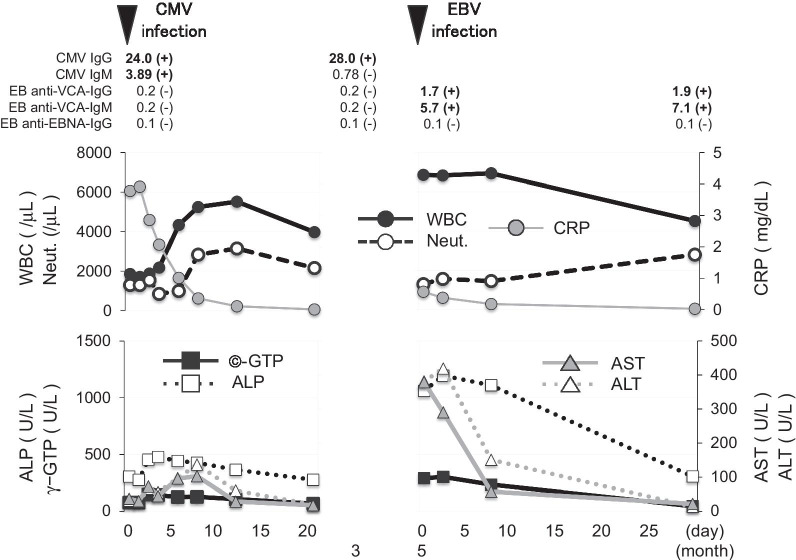
Clinical time course in this patient. Decreased WBC and increased CRP levels were observed after CMV infection, and mild liver dysfunction was also detected. The data for various virus antibodies showed a primary CMV pattern, and EBV antibody was negative at that time. About 5 months later, she had primary EBV infection. She had very severe liver dysfunction after EBV infection compared with after CMV infection. *CMV* *Cytomegalovirus*, *EBV* Epstein–Barr virus, *WBC* white blood cell, *CRP* C-reactive protein, γ-*GTP* γ-glutamyl transpeptidase, *ALP* alkaline phosphatase, *AST* aspartate aminotransferase, *ALT* alanine aminotransferase

A 20-year-old healthy Japanese woman had a 2-day history of high fever and consulted the emergency room in Kawasaki Medical School. There was no significant past medical history except for mycoplasma pneumonia at the ages of 1 and 18. She had sex for the first time 6 months earlier. She had erythema in the pharynx and lymph node enlargement at the posterior cervical and left axillary region, but there were no findings on physical and neurological examination. Her chest sounds were clear, and she had no cough. The tips of the liver and spleen were not palpable. Her vital signs were as follows: heart rate 108 beats/minute, blood pressure 101/58 mmHg, and temperature 38.9°C. Table 1 shows the laboratory data on admission. Leukocytopenia and increased C-reactive protein (CRP) were observed: white blood cell (WBC) count, 1710 /μL (neutrophils 76.0%; monocytes 5.0%; lymphocytes 18.0%; atypical lymphocytes 1.0%); CRP, 3.92 mg/dl. She had mild liver dysfunction: γ-GTP, 78 U/L; alkaline phosphatase (ALP), 276 U/L; aspartate aminotransferase (AST), 30 U/L; alanine aminotransferase (ALT), 28 U/L. Her chest X-ray and computed tomography (CT) on admission revealed no remarkable change including pneumonia, and her abdominal CT revealed slight hepatomegaly and splenomegaly. Neither influenza A nor B antigens were detected, and *Mycoplasma pneumonia* antigen was not detected. Her hepatitis B surface antigen and hepatitis C antibody tests were negative. Human immunodeficiency virus (HIV) antigen/antibody test was negative (cutoff index <0.3). Her *Treponema pallidum* hemagglutination (TPHA) was negative. Pathogenic bacteria were not detected, although small amounts of *Corynebacterium sp.* and Gram-positive cocci were detected in urine culture. We started antibiotic therapy (500 mg/day of levofloxacin), because we failed to rule out a bacterial infection due to an elevated CRP level. She was suspected of having a bladder infection by her previous doctor, and in fact she had bacteriuria. Her virus antibodies were as follows: EBV (EBV) anti-viral capsid antigen (VCA) immunoglobulin M (IgM) antibody, 0.2 (−) (fluorescent antibody method [FA], SRL Inc., Tokyo); EBV anti-VCA immunoglobulin G (IgG) antibody, 0.2 (−) (FA, SRL Inc., Tokyo); EBV anti-EBV nuclear antigen (EBNA) Immunoglobulin (Ig) G antibody, 0.1 (−) (FA, SRL Inc., Tokyo); CMV IgM antibody, 3.89 (+) (enzyme immunoassay (EIA), SRL Inc., Tokyo); CMV IgG antibody, 24.0 (+) (EIA, SRL Inc., Tokyo). Based on these findings, we finally arrived at a diagnosis of primary CMV infection. Four days later, her temperature was reduced to under 37°C, and about 1 week later, her clinical symptoms disappeared and laboratory data including leukocytopenia and liver dysfunction were improved (Fig. 1). She was discharged 10 days after admission. She did not experience another abnormal physical condition after discharge, but we continued to observe her condition on an outpatient basis. Therefore, we checked her virus antibodies. Her CMV IgM antibody became negative about 3 months later, and we confirmed that her CMV infection was a prior infection pattern.

**Table 1:** Clinical data after primary *Cytomegalovirus* infection in this subject

Variable	Result	Reference range	Variable	Result	Reference range
*Peripheral blood*			*Blood biochemistry*		
White blood cells (/μL)	1710	3300–8600	Total protein (g/dL)	6.3	6.6–8.1
Neutrophils (%)	76.0	52.0–80.0	Albumin (g/dL)	3.6	4.1–5.1
Eosinophils (%)	0.0	1.0–5.0	Globulin (g/dL)	2.7	2.2–3.4
Basophils (%)	0.0	0.0–1.0	Cholinesterase (U/L)	163	240–486
Monocytes (%)	5.0	1.0–6.0	Total bilirubin (mg/dL)	2.7	0.4–1.5
Lymphocytes (%)	18.0	20.0–40.0	Direct bilirubin (%)	11	30–52
Atypical lymphocytes (%)	1.0		AST (U/L)	30	13–30
Red blood cells (×10^4^/μL)	429	435–555	ALT (U/L)	28	10–42
Hemoglobin (g/dL)	12.8	13.7–16.8	LDH (U/L)	221	124–222
Platelets (×10^4^/μL)	8.7	15.8–34.8	ALP (U/L)	276	106–322
*Congealing fibrinogenolysis system*			γ-GTP (U/L)	78	13–64
PT (seconds)	14.3	9.3–12.5	BUN (mg/dL)	8	8–20
PT-INR	1.25	0.85–1.13	Creatinine (mg/dL)	0.69	0.65–1.07
PT activity (%)	66.5	80.7–125.2	Uric acid (mg/dL)	5.2	2.6–5.5
APTT (seconds)	46.7	26.9–38.1	Amylase (U/L)	89	44–132
Fibrinogen (mg/dL)	263	160–380	Creatinine kinase (U/L)	62	41–153
Urinary test			Plasma glucose (mg/dL)	91	
Urinary pH	7.0	5.0–7.5	Total cholesterol (mg/dL)	168	142–248
Urinary protein	±	−	LDL cholesterol (mg/dL)	89	65–139
Urinary sugar	−	−	HDL cholesterol (mg/dL)	57	40–90
Urinary ketone body	−	−	Triglyceride (mg/dL)	71	40–149
Urinary blood	±	−	Sodium (mmol/L)	135	138–145
Urinary bacteria	±	−	Potassium (mmol/L)	3.7	3.6–4.8
			Chloride (mmol/L)	102	101–108
			CRP (mg/dL)	3.92	< 0.14
			Procalcitonin (ng/mL)	0.39	0.00–0.05

*PT* prothrombin time, *INR* international normalized ratio, *APTT* activated partial thromboplastin time, *AST* aspartate aminotransferase, *ALT* alanine aminotransferase, *LDH* lactate dehydrogenase, *ALP* alkaline phosphatase, *γ-GTP* γ-glutamyl transpeptidase, *BUN* blood urea nitrogen, *LDL* low-density lipoprotein, *HDL* high-density lipoprotein, *CRP* C-reactive protein

About 5 months later, she again experienced fever and lymph node enlargement at the posterior cervical region and visited our emergency room. She had no symptoms other than high fever and lymph node enlargement, and there were no findings on physical or neurological examination. Her vital signs were as follows: heart rate 85 beats/minute, blood pressure 98/56 mmHg, and temperature 38.9°C. Table 2 shows the laboratory data at that time. Atypical lymphocytes were detected and the CRP level was slightly increased: WBC count, 6860/μL (neutrophils 19.0%; monocytes 6.0%; lymphocytes 73.0%; atypical lymphocytes 2.0%); CRP, 0.57 mg/dl. Furthermore, she had severe liver dysfunction: γ-GTP, 291 U/L; ALP, 1060 U/L; AST, 379 U/L; ALT 381 U/L. We thought that it would be better to hospitalize her and start drip infusion of monoammonium glycyrrhizinate glycine for severe liver dysfunction, but she did not agree to the hospitalization. Therefore, instead we started 60 mL/day of monoammonium glycyrrhizinate glycine on an outpatient basis. At that time, we suspected viral infection and thus we did not administer antibiotics. She visited our office for drip infusion of monoammonium glycyrrhizinate glycine every day, and about 5 days later the high fever disappeared. About 1 week later, her clinical symptoms and laboratory data were improved (Fig. 1) and we stopped the therapy with monoammonium glycyrrhizinate glycine. At that time, her EBV antibodies were as follows: EBV anti-VCA IgM antibody, 1.7 (+); EBV anti-VCA IgG antibody, 5.7 (+); EBV anti-EBNA IgG antibody, 0.1 (−). Therefore, we finally diagnosed her condition as primary EBV infection.

**Table 2:** Clinical data after primary Epstein–Barr virus infection in this subject

Variable	Result	Reference range	Variable	Result	Reference range
*Peripheral blood*			*Blood biochemistry*		
White blood cells (/μL)	6860	3300–8600	Total protein (g/dL)	7.4	6.6–8.1
Neutrophils (%)	19.0	52.0–80.0	Albumin (g/dL)	4.0	4.1–5.1
Eosinophils (%)	0.0	1.0–5.0	Globulin (g/dL)	3.4	2.2–3.4
Basophils (%)	0.0	0.0–1.0	Cholinesterase (U/L)	182	240–486
Monocytes (%)	6.0	1.0–6.0	Total bilirubin (mg/dL)	1.9	0.4–1.5
Lymphocytes (%)	73.0	20.0–40.0	AST (U/L)	379	13–30
Atypical lymphocytes (%)	2.0		ALT (U/L)	381	10–42
Red blood cells (×10^4^/μL)	686	435–555	LDH (U/L)	650	124–222
Hemoglobin (g/dL)	13.6	13.7–16.8	ALP (U/L)	1060	106–322
Platelets (×10^4^/μL)	15.5	15.8–34.8	γ-GTP (U/L)	291	13–64
*Congealing fibrinogenolysis system*			BUN (mg/dL)	10	8–20
PT (seconds)	11.9	9.3–12.5	Creatinine (mg/dL)	0.62	0.65–1.07
PT-INR	1.03	0.85–1.13	Uric acid (mg/dL)	4.8	2.6–5.5
PT activity (%)	94.9	80.7–125.2	Plasma glucose (mg/dL)	140	
APTT (seconds)	39.6	26.9–38.1	Total cholesterol (mg/dL)	182	142–248
Fibrinogen (mg/dL)	229	160–380	Sodium (mmol/L)	137	138–145
			Potassium (mmol/L)	3.9	3.6–4.8
			Chloride (mmol/L)	101	101–108
			CRP (mg/dL)	0.57	< 0.14

*PT* prothrombin time, *INR* international normalized ratio, *APTT* activated partial thromboplastin time, *AST* aspartate aminotransferase, *ALT* alanine aminotransferase, *LDH* lactate dehydrogenase, *ALP* alkaline phosphatase, *γ-GTP* γ-glutamyl transpeptidase, *BUN* blood urea nitrogen, *CRP* C-reactive protein

In addition, we checked the antibodies for both CMV and EBV infection several times in this patient, and Table 3 shows the time course for these antibodies. Such time course clearly shows that this patient had primary EBV infection shortly after primary CMV infection.

**Table 3:** Time course of various antibodies for both CMV and EBV in this patient

Virus antibody	Just after primary CMV infection	3 months after CMV infection	5 months after CMV infection and just after primary EBV infection	6 months after CMV infection	Reference range
CMV IgG antibody	24.0 (+)	28.0 (+)	N/A	N/A	< 0.2
CMV IgM antibody	3.89 (+)	0.78 (−)	N/A	N/A	< 0.80
EBV anti-VCA IgG antibody	0.2 (−)	0.2 (−)	1.7 (+)	1.9 (+)	< 0.5
EBV anti-VCA IgM antibody	0.2 (−)	0.2 (−)	5.7 (+)	7.1 (+)	< 0.5
EBV anti-EBNA IgG antibody	0.1 (−)	0.1 (−)	0.1 (−)	0.1 (−)	< 0.5

*CMV* *Cytomegalovirus*, *EBV* Epstein–Barr virus, *VCA* viral capsid antigen, *EBNA* EBV nuclear antigen, *N/A* not applicable

## Discussion

Herein, we report a healthy adult Japanese woman with primary EBV infection shortly after primary CMV infection. It is difficult to diagnose such infectious diseases based only on subjective symptoms such as a high fever, pharyngitis and lymphadenopathy. IM and MLI are major illnesses which are caused by EBV and CMV, respectively, and are often accompanied by such symptoms [2, 9]. Adults with primary EBV or CMV infection often experience serious symptoms, although such infection in childhood is generally subclinical with either virus. Our patient had almost the same symptoms, including high fever, pharyngitis and lymphadenopathy, after both CMV and EBV infection. However, she had decreased WBC and high CRP after CMV infection. In contrast, WBC and CRP levels were not altered after EBV infection. In addition, she had very severe liver dysfunction after EBV infection compared with after CMV infection. Some reports have compared subjects with EBV infection and those with CMV infection. However, it is very interesting to compare subjective symptoms and/or clinical data after EBV and CMV infection in the same patient within a short period of time. In general, CMV infection affects patients who are 10–15 years older and who have milder lymphadenopathy and pharyngitis compared with EBV infection, but more frequent and serious hepatitis is observed after CMV infection [3–5]. In clinical practice, however, it is very difficult to distinguish EBV infection from CMV infection without checking various antibodies for these viruses. Indeed, our patient was not diagnosed based only on subjective symptoms, physical examination and laboratory data without tests of such virus-related antibodies.

It has been reported that in industrialized societies, individuals with lower socioeconomic status are more easily infected with EBV and at younger ages than those in affluent groups [10]. It is also well known that EBV and CMV are common opportunistic infections in immunocompromised conditions such as HIV infection, but not in healthy adult subjects [11]. In addition, co-infection with EBV and CMV has been reported mainly in children [12, 13]. However, it is difficult to determine which findings (e.g. liver dysfunction) are mainly due to EBV and/or CMV infection. Therefore, it is interesting to compare such symptoms and/or clinical data in the same patient. Also, it was pointed out recently that both EBV and CMV antibody positivity rates are lower in Japanese healthy adults. Both EBV and CMV infection are common in Japan, although in most cases they remain latent throughout human life. It was previously reported that many Japanese were infected with EBV in infancy as subclinical infection and with CMV in the perinatal period as subclinical infection. However, the age of first infection of EBV and CMV has recently clearly been increasing. In addition, it seems that the first infection of EBV and CMV would be increased in the future, although the reported cases are few and the case of overt infection is rare. Such phenomena have attracted much attention, and we are very concerned that the decrease in such rates would lead to an increase in EBV and/or CMV infection in the future.

There are limitations in this case report. First, we did not perform EBV and CMV polymerase chain reaction (PCR), and the diagnosis was based mainly on the serology data. Distinguishing current infection from past infection is more commonly and more easily accomplished through virus antibody tests [14]. Our patient happened to have two infections; the primary EBV infection manifested shortly after the primary CMV infection. Since the PCR method confirms current active infection [15], in this case, the serology method would have made it easier to understand her pathology. Second, there are some reports of cross-reactivity and false positivity and negativity [16]. Both EBV and CMV belong to the herpes virus group, and IgM-type antibodies of CMV are detected in about one-third of cases of initial EBV infection [17, 18]. While it is known that cross-reactivity and false positivity and negativity cause problems in patients with two infections at the same time, there was some time lag (about 5 months) between the two infections in this case. Therefore, we think that there is no problem with cross-reactivity or false positivity/negativity in this case. Furthermore, in her first virus infection, her antibodies showed a primary CMV infection pattern [CMV IgM antibody, (+); CMV IgG antibody, (+)], although her EBV infection antibody was all negative. However, in her second virus infection, her antibodies showed a primary EBV infection pattern [EBV anti-VCA IgM antibody, (+); EBV anti-VCA IgG antibody, (+); EBV anti-EBNA IgG antibody, (–)]. If her primary EBV infection and primary CMV infection manifested at the same time, as was the case in previous reports [11, 12], cross-reactivity and false positivity/negativity would have definitely remained controversial. Third, we did not check the patient's partner for antibodies to EBV and CMV, but since he was older than the patient, we assume that he was already infected with both EBV and CMV, just as the average Japanese are already infected.

## Conclusions

We should bear in mind that primary EBV infection and/or primary CMV infection is possible when patients have symptoms such as high fever, pharyngitis and lymphadenopathy, even in healthy adults.

## Data Availability

Not applicable.
